# Wound healing after radiation therapy: Review of the literature

**DOI:** 10.1186/1748-717X-7-162

**Published:** 2012-09-24

**Authors:** Frank Haubner, Elisabeth Ohmann, Fabian Pohl, Jürgen Strutz, Holger G Gassner

**Affiliations:** 1 Department of Otorhinolaryngology, Division of Facial Plastic Surgery, University of Regensburg, Regensburg, Germany; 2 Department of Radiotherapy, University of Regensburg, Regensburg, Germany

**Keywords:** Wound healing, Radiation therapy

## Abstract

Radiation therapy is an established modality in the treatment of head and neck cancer patients. Compromised wound healing in irradiated tissues is a common and challenging clinical problem. The pathophysiology and underlying cellular mechanisms including the complex interaction of cytokines and growth factors are still not understood completely. In this review, the current state of research regarding the pathomechanisms of compromised wound healing in irradiated tissues is presented. Current and possible future treatment strategies are critically reviewed.

## Introduction

**Figure 1:**
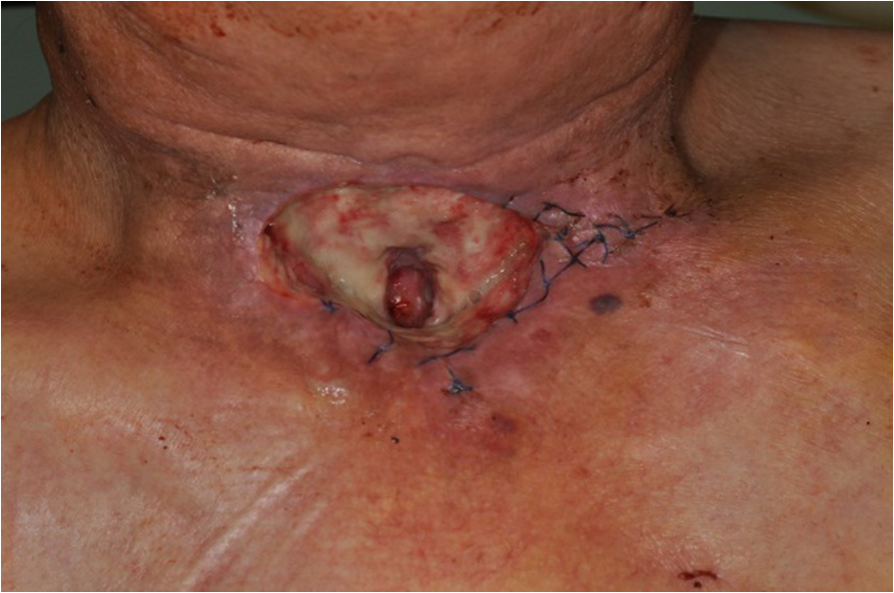
**Clinical case of a 55-year-old male, six months after primary radiochemotherapy due to an advanced squamous cell carcinoma of the hypopharynx.** Skin atrophy and soft tissue necrosis were observed 8 weeks after the completion of therapy.

Radiation is employed as neoadjuvant, primary and adjuvant therapy for head and neck cancer. Complications after radiation therapy occur in up to 60 percent of surgical patients. Clinical sequelae include skin atrophy, soft tissue fibrosis, desquamation, epithelial ulceration, fistula formation and major vessel rupture [1,2]. Impaired peri- and postoperative wound healing and the complications associated with it can be observed [3,4] frequently and may require extensive reconstructive efforts [5-7] (Figure 1).

## Physiological wound healing

**Figure 2:**
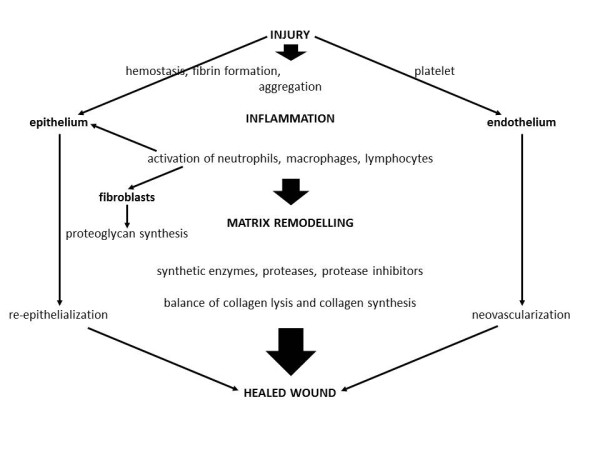
**Schematic concept of wound healing. Adapted from Hunt TK **[8].

Adequate wound healing involves interactions of cells. Cell biologic mechanisms relevant to the process include interaction of keratinocytes, fibroblasts and endothelial cells [8]. Epithelial closure of a wound is an important aspect of this complex biological process and relies primarily on the concerted action of activated keratinocytes and dermal fibroblasts [9]. Three phases of wound healing with distinctive biochemical profiles have been described (Figure 2).

Hemostasis and inflammation (phase 1, day 0 to 4), are followed by proliferation (phase 2, day 3 to week 3) and maturation (phase 3, week 3 to 2 years) [10,11]. These three phases are regulated by a complex network of interacting cytokines, growth factors and their cellular receptors.

## Effects of radiation therapy on wound healing

Wound healing occurs in an ordered sequence of cellular interactions. Repetitive radiation injury disrupts this highly organized sequence of events, resulting in repetitive inflammatory responses and ongoing cellular regeneration [12].

There is an important distinction to be made between the early and the late side-effects of radiation therapy: Early side effects include erythema, dry desquamation, hyperpigmentation and hair loss [13]. Late effects include skin atrophy, dryness, telangiectasia, dyschromia, dyspigmentation, fibrosis, and ulcers [14].

The inflammatory and proliferative phases may be disrupted by the early effects of radiation. Affected factors during the inflammatory phase include transforming growth factor beta (TGFβ), vascular endothelial growth factor (VEGF), tumor necrosis factor-α (TNF-α), interferon-γ (IFN- γ) and proinflammatory cytokines such as interleukin-1 and interleukin-8 [12]. These cytokines are overexpressed after the radiation injury leading to uncontrolled matrix accumulation and fibrosis [15].

The proliferative phase is characterized by granulation tissue formation, re-epitheliaziation and neovascularization. This phase is mainly regulated by TGFβ, VEGF, epidermal growth factor (EGF), fibroblast growth factor (FGF) and platelet-derived growth factor (PDGF) [12]. Nitric oxide (NO) promotes wound healing by an induction of collagen deposition [16]. NO levels have been reduced in irradiated wounds of experimental animals [17]. This finding may explain the impaired strength of irradiated wounds.

During the remodeling phase, matrix metalloproteinases (MMP) and their tissue inhibitors are central to the process [18,19]. MMP-1 is decreased after radiation therapy, which may contribute to inadequate soft tissue reconstitution [19] (Table 1).

**Table 1:** Possible key factors affected by radiotherapy with respect to the phases of wound

**Phase of wound healing**	**Factors affected by radiation therapy**
**Inflammation**	TGFβ, VEGF, interleukin-1, interleukin-8, TNFα, IFN-γ
**Proliferation**	TGFβ, VEGF, EGF, FGF, PDGF, NO
**Remodelling**	MMP-1, MMP-2, MMP-12, MMP-13, TIMP

Wound healing factors affected by radiation therapy.

Keratinocytes represent a crucial cell type in the repair of late epithelial wounds and ulcers. Multiple molecular biological changes are observed in this cell after radiation when compared to radiation-naïve skin. In human radiogenic wounds, these cells show a shift in the expression from high molecular keratins 1 and 10 to the low molecular keratins 5 and 14. In non-healing ulcers, keratinocytes display a decreased expression of transforming growth factor-alpha and –beta(1), fibroblast growth factor 1 and 2, keratinocyte growth factor, vascular endothelial growth factor, and hepatocyte growth factor. Expression of the matrix metalloproteinases 2, 12 and 13 has been shown to be elevated in irradiated human keratinocytes and fibroblasts [20].

Fibroblasts play the central role in wound healing through deposition and remodeling of collagen fibers. In irradiated tissue, fibroblasts have been shown to generate a disorganized deposition of collagen bundles. One likely mechanism resulting in disorganized collagen deposition is dysregulation of MMP and TIMP. These enzymes regulate extracellular matrix synthesis [21,22]. As TGF-beta in turn regulates MMPs and TIMPs, this cytokine may be of particular relevance to radiogenic ulcers.

## Current strategies in treating irradiated wounds

Established strategies in treating radiogenic ulcers with delayed and inadequate healing include standard wound care, vacuum-assisted devices, substitution of nutritional deficiencies, and measures to optimize blood and oxygen supply [6,23].

Hyperbaric oxygen seems to optimize the partial pressure of tissue oxygen. It is frequently used for the treatment of osteoradionecrosis [24-27]. The postulated effects include an increased capillary density and more complete neovascularization [28-33]. Kendall et al. tested hyperbaric oxygen in a cell culture model and found that its application resulted in the downregulation of 9 genes involved in adhesion, angiogenesis, inflammation and oxidative stress [34]. Interleukin-8 mRNA levels were also suppressed after daily exposure of hyperbaric oxygen to endothelial cells in this study [34]. IL-8 is one key factor in the inflammatory phase of wound healing. This suggests that the benefit of hyperbaric oxygen may not be limited to osteoradionecrosis and further investigations regarding additional therapeutic effects may be valuable.

Currently, hyperbaric oxygen therapy is clinically used in chronic diabetic ulcers and wound healing complications after radiotherapy. Its efficacy has been proven by several randomized trials, but it is it is important to emphasize that for both diabetic wounds and radiation injuries, this therapy is used in conjunction with standard wound care management techniques [35]. A possible new approach for the hyperbaric oxygen therapy is the treatment of dermal wounds after flap surgery. Multiple case reports and animal studies have been written on this issue. Prospective clinical trials are necessary to support the application of hyperbaric oxygen to improve flap survival [36].

## Future aspects in the treatment of irradiated wounds

Research into new therapeutic approaches to treat radiogenic ulcers includes: Special dressings, injection of (multipotent) cells, topical administration of active substances and the use of growth factors (Table 2).

**Table 2:** Current experimental strategies in the treatment of irradiated wounds

**Experimental strategies**			
**Dressings**	**Injection of cells**	**Topicals**	**Application of growth factors**
hydrogel membranes	unirradiated fibroblasts	polyphenolic bioflavonoids	TGF-beta (1,2,3) and antisense TGF-beta oligonucleotides
bacterial cellulose	dermis-derived multipotent cells	phenylbutyrate, valproic acid, ascorbic acid	platelet-derived growth factor
silver nanoparticles	adipose-derived stem cells	tissue and microbial transglutaminases	recombinant human epidermal growth factor
skin allografts		copper tripeptide	macrophage colony stimulating factor
		Thrombin receptor-activating peptide	granulocyte colony stimulating factor
			basic fibroblast growth factor
			vascular endothelial growth factor

Overview on current experimental strategies.

### Special dressings

Different types of membranes and other covers of radiogenic skin injuries have been developed in the past.

### Hydrogel membranes

Hydrogel membranes have a stable, flexible and transparent structure. Data of Lu et al. indicate that the hydrogel membranes act as a semiocclusive dressing and maintain a moist environment over the wound bed. Through this mechanism, re-epithelialization is thought to be enhanced because of the accumulation of cytokines and growth factors that support wound healing [37]. The use of hydrogels for partial thickness wounds after burns or laser resurfacing is supported by case reports [38]. Prospective, controlled data on their treatment effect in radiogenic wounds are not available.

### Silver membranes

Bacterial cellulose impregnated membranes and poviargol, an antiseptic substance containing silver, was shown to accelerate the healing of irradiated wounds in an animal model [39]. A recent animal study has revealed that silver nanoparticles have the potential to promote wound healing through accelerated re-epithelialization and enhanced differentiation of fibroblasts. The exact effect of silver-coated membranes on skin repair is only partially understood. Hypothetic mechanisms include the fact that collagen bundle organization and the tensile properties of the skin [40] are found to be improved.

### Skin allografts

A human deceased-donor skin allograft provides dermoprotection and promotion of re-epithelialization. It is utilized until autografting is possible or re-harvesting of donor sites becomes available [41]. This treatment is currently limited to burn injuries. It may be hypothesized that similar effects may be achieved in radiogenic ulcers, but clinical data and trials with sufficient numbers are not available.

### Injection of cells

Wound healing is a complex mechanism that requires active cellular interactions. These interactions are only possible if a sufficient number of intact and healthy cells are present. Radiation leads to impaired cellular activity and cell death. Injection of cells has the potential of enhancing wound healing in irradiated tissues. Nevertheless, there is a significant risk of infection and possible tumor induction by injecting viable cells in vivo. All of the studies discussed below have to be seen in this context. All interventions are experimental and presently not applied in routine clinical practice.

### Autologous fibroblasts

Autologous, unirradiated fibroblasts were shown in a rat model by Ferguson et al. to significantly improve healing of the irradiated surgical wound. This was evidenced by injections of culture medium, irradiated fibroblasts and non-irradiated fibroblasts in previously irradiated surgical wounds. The autologous non-irradiated fibroblasts caused greater increases of breaking load and ultimate tensile strength of the wounds than the irradiated control cells [42]. Other authors also report that implanting isolated dermal fibroblasts leads to a significant increase of wound strength in irradiated mice skin. This effect may rescue wounds from the otherwise irreversible effect of prior irradiation [43]. Both studies show promise for the treatment of irradiated wounds in patients. The injection of autologous fibroblasts would be possible at the end of a surgical procedure in irradiated patients. But this method has not yet led to the conduction of human clinical trials. One problem in this context is the risk of inducing neoplastic lesions by injecting viable cells.

### Multipotent stem cells

Dermis-derived multipotent stem cells seem to be easily harvested from animal skin.

Chunmeng reports that both topical and systemic transplantation of dermis-derived multipotent cells accelerates healing after a radiation-induced injury [44]. The suggested mechanism is the synthesis of important wound healing factors such as VEGF, PDGF and TGF-beta by these cells. An interesting finding of this animal study on rats was also the analysis of the cell supernatants. A medium previously incubated with dermis-derived stem cells seems to contain relevant amounts of wound-healing promoting factors such as VEGF, PDGF and TGF-beta. This presents a valuable approach for further clinical studies.

Human mesenchymal stem cells have also been discussed in the therapy of radiogenic ulcers. Adipose-derived stem cells have been described for the therapy of limited local injuries and seem to improve angiogenesis and the reconstitution of dermal architecture [45].

Akita et al. describe the use of adipose-derived stem cells (ADSC) in a female suffering from a radiogenic wound 40 years after irradiation due to a uterine carcinoma. They used an artificial dermis impregnated with ADSC to cover the wound bed and injected ADSC into the margins. This method was combined with the local administration of a basic fibroblast growth factor to improve angiogenesis. The authors documented excellent results of wound healing and a durable regenerated tissue after 1.5 years [46]. The results of this study may be open to various interpretations. The observed effect may be due to the adipose-derived stem cells, the artificial dermis or the application of growth factors. Combined or additive effects cannot be excluded to explain the success of this approach. Clinical studies investigating the effect of adipose-derived stem cell injections into radiogenic wounds are not available. Hadad et al. developed a wound-healing model to study such effects in pigs [47]. They found no effect of the ASCs injections alone, but they could document improved wound healing by a combination therapy of ASC and platelet rich plasma injections into irradiated wounds. The most important effects were an accelerated wound closure and an increased microvessel density after the combined treatment.

A proangiogenetic effect of stem cell injections has been suggested by the authors, but other mechanisms may also serve to explain the findings in this study. New vessel formation and the optimizing of tissue microcirculation present a valuable effect of stem cell injections with respect to cutaneous wound healing. But the benefit on wound healing might end up providing a favorable environment for tumor recurrence after radiotherapy. High risk constellations or the application of stem cell injections in elderly people who have a diminished life time may put these patients at risk for developing tumor recurrence, which would justify further clinical trials. The clinical observations of Akita et al. are supported by another porcine study. Porcine skin seems to react similarly to human skin as far as radiogenic injuries are concerned. Also wound-healing mechanisms are similar in humans and pigs [48]. That is why porcine animal studies are of major value in this field of research. Forcheron et al. injected autologous adipose-derived stem cells into the skin of pigs irradiated with 50Gy [49]. This study focused on the cutaneous radiation syndrome. In this context the authors observed an improved clinical wound healing and an enhanced re-epitheliasation in animals injected with adipose-derived stem cells.

The antioxidant effects of adipose-derived stem cells [50] are reported by Kim et al. These effects seem to be mainly mediated through the activation of dermal fibroblasts and keratinocytes via the paracrine mechanism.

Another advantage of adipose-derived stem cells over other stem cell sources is that they are easily obtained in large quantities by liposuction. Also their potency to synthesize growth factors and cytokines shows promise for the use in skin repair and regeneration [51,52]. A cell culture study by Lee et al. supports the stimulatory effects of adipose-derived stem cells on cutaneous wound healing [53]. In their study, the proliferation of fibroblasts and their collagen synthesis were increased by a conditioned medium of adipose-derived stem cells in vitro.

The studies mentioned above reveal that adipose-derived stem cells seem to interact directly and via paracrine mechanisms with the key cells of wound healing. Clinical trials analyzing the role of these cells and their combination with other wound-healing promoting factors would contribute to a better understanding of the potential value of this treatment regimen.

### Experimental application of active agents

The topical application of active agents to reduce the side effects of radiation therapy was analyzed in several cell culture and animal studies.

### Bioflavonoids

Biologically active polyphenolic bioflavonoids have been shown in cell culture studies with keratinocytes to have positive effects on angiogenesis. The suggested mechanism is an increased expression of the vascular endothelial growth factor via the TNF (tumor necrosis factor)-alpha-pathway [54]. Angiogenesis plays a central role in wound healing. VEGF is believed to be the most prevalent, efficacious and long-term signal that is known to stimulate angiogenesis in wounds.

### Histone deacetylase inhibitors

Histone deacetylase inhibitors (phenylbutyrate, trichostatin A and valproic acid) were shown to suppress the cutaneous radiation syndrome in rat skin. Chung et al. analyzed different histone deacetylase inhibitors with respect to acute skin reaction and dermal fibrosis. They used vaseline as a negative control and performed immunohistological analyses. The underlying mechanism seems to be the suppression of the aberrant expression of radiation-induced transforming growth factor-beta 1 and 2 and tumor necrosis factor alpha [55].

This study presents the value of antitumor histone deacetylase inhibitors which may suppress cutaneous radiation syndrome and are possible new agents for increasing therapeutic gain in cancer radiotherapy.

### Ascorbic acid

Ascorbic acid has been shown to result in the significant acceleration of healing of radiogenic ulcers. This was shown in a mouse model with doses of 10,16 and 20 Gy. A full thickness skin wound was created after radiation. Histological evaluations were performed at various times after wounding. Pretreatment with ascorbic acid augmented the synthesis of collagen significantly as revealed by an increase in hydroxyproline content. In the ascorbic acid group, an earlier wound closure was observed compared to the control group [56]. Optimizing collagen synthesis in a poorly healing wound by the application of ascorbic acid seems to be reasonable, but clinical trials are still lacking.

### Copper tripeptide

Copper tripeptide accelerates the growth of normal and irradiated fibroblasts, which was shown in a cell culture study by Pollard et al.:

Primary human dermal fibroblasts were explanted from intraoperative specimens obtained from patients after radiation therapy for head and neck cancer. Normal unirradiated fibroblasts served as a control. Irradiated fibroblasts treated with copper tripeptide showed a similar proliferation rate as the untreated controls, and produced significantly more basic fibroblast growth factor and vascular endothelial growth factor than untreated controls. An early increase in growth factors and cell proliferation by irradiated fibroblasts treated with copper tripeptide may improve wound healing [57].

### Thrombin receptor-activating peptide

Thrombin receptor-activating peptide (P517-30) has been shown to increase wound-breaking strength in irradiated tissues. This substance is a synthesized high-affinity thrombin receptor binding peptide. In a rat model, Cromack et al. revealed that P517-30 directly stimulates resident endothelial cells and fibroblasts to overcome dermal and circulating monocytic deficits. These results suggest a mechanism to accelerate wound healing with a potential clinical application and emphasize the activity of thrombin as a growth factor [58]. Special hydrogels could be possible applications for these growth factors [59].

### Application of growth factors

Different growth factors have been analyzed for their potential role in wound healing, albeit their carcinogenic potential must be taken into consideration.

Important factors that may be suitable for this therapy include recombinant human granulocyte colony-stimulating factor (rhG-CSF), recombinant human macrophage colony-stimulating factor (rhM-CSF), basic fibroblast growth factor, TGF-beta and an inhibitor of transforming growth factor (TGF)-beta(1) receptor kinase [60-65].

### TGF-beta and TGF-beta antisense oligonucleotides

The exogenous application of TGF-beta (1) and (3) has the potential to act as a radioprotective agent, especially in adjuvant therapeutic regimens. In a rat model, reduced TGF-beta(3) expression was observed in the irradiated graft bed and induction of collagen synthesis was observed after application of TGF-beta (1) [66,67].

Whether these findings are relevant in the clinical setting after radiation therapy remains to be seen, because TGF-beta suppression seems to have favorable effects as well:

TGF-β is a strong stimulator of extracellular matrix deposition. A new pharmacological approach was the development of the TGF-beta antisense oligonucleotide technology. This intervention shows promise as a therapeutic option for the inhibition of proteolytic tissue destruction, which is one key approach to optimize wound healing. Irradiated wounds often fail to heal because of high amounts of MMPs. TGF-beta antisense oligonucleotides seem to affect the major cell types of dermal wound healing: Fibroblasts, keratinocytes and endothelial cells were influenced in their gene expression of MMPs. MMP-1 and MMP-9 were significantly decreased after the treatment of fibroblasts and keratinocytes with TGF-beta antisense oligonucleotides in vitro [68]. Animal models of wound healing and scarring after eye surgery documented an anti-scarring effect of the TGF-beta antisense oligonucleotides in vivo [69].

TGF-beta antisense oligonucleotide technology shows promise. VEGF up-regulation was observed in vitro and a pro-angiogenetic effect of TGF-beta antisense oligonucleotides in radiation-induced dermal wounds was suggested [70]. Another study showed TGF-beta antisense oligonucleotides result in an increased expression of MMP protein and mRNA in tissue samples from radiation-induced chronic dermal wounds when compared to normal human skin. These effects were observed by immunohistochemistry and microarray analysis. Antisense TGF-beta oligonucleotide treatment also significantly down-regulates MMP secretion in vitro. Through these mechanisms, proteolytic tissue destruction may be inhibited in radiogenic ulcers [71]. Clinical trials would be helpful to better understand the effects of TGF-beta oligonucleotides in vivo. The interesting issue in this context is balancing the positive effects of matrix formation by TGF-beta and the negative side effects of a TGF-beta overexpression after radiotherapy.

### Platelet-derived growth factor

Platelet-derived growth factor (PDGF) may be useful as a topical agent in post-irradiation surgical incisions [47,72]. PDFG is already in clinical use and shows favorable results. In previously irradiated tissue, rhPDGF (recombinant human platelet-derived growth factor) has been shown to enhance wound healing through the induction of granulation tissue formation. PDGF is a cytokine that is only activated in the presence of bone marrow-derived cells like wound macrophages. The transformation of wounds from a chronic to a short-term healing state after rhPDGF treatment was documented by serial histological examinations. The risk of tumor induction is difficult to quantify and must be considered prior to clinical application [73,74].

### Granulocyte macrophage-colony stimulating factor

Granulocyte macrophage-colony stimulating factor (GM-CSF) has been shown to modulate lipid peroxidation and glutathione content in skin wounds [75].

It has been demonstrated that GM-CSF increases the number of neutrophils, eosinophils and monocytes with corresponding bone marrow changes and might be suitable for different approaches in cancer therapy [76]. Irradiation decreases incisional healing and produces oxygen radicals that damage cells. Because of the lipid component in the membrane, the cellular membranes are particularly susceptible to radiation damage due to peroxidation. Glutathione acts as a co-substrate in the enzymatic repair of radiation damage. Suppressed levels of glutathione have been shown to increase after the administration of granulocyte macrophage-colony stimulating factor in irradiated rats. The reduction of complications associated with radiochemotherapy is one possible goal of GM-CSF application.

### Recombinant human epidermal growth factor (rhEGF)

The use of recombinant human epidermal growth factor (rhEGF) was studied in mice after radiation with 45 Gy. Histological examinations showed that treatment with EGF accelerated normal wound healing when compared to no treatment. Collagen distribution was significantly increased in the group treated with EGF. Dermal and epidermal structure was also more stable in the treatment group [77].

### Combined treatments

Pollard et al. investigated cytokine expression in a cell culture model of irradiated human skin isolates. Basic fibroblast growth factor and vascular endothelial growth factor were found to be less expressed in the radiation-induced dermal wounds [78]. These authors concluded that an early combined increase in basic fibroblast growth factor and vascular endothelial growth factor production in irradiated fibroblasts may improve wound healing [57]. Follow-up clinical studies have not yet been conducted.

There is some evidence to support the combination of different growth factors to escalate their therapeutic effect [79]. Important factors that may be suitable for combination therapy include recombinant human granulocyte colony-stimulating factor (rhG-CSF), recombinant human macrophage colony-stimulating factor (rhM-CSF), basic fibroblast growth factor and an inhibitor of transforming growth factor (TGF)-beta(1) receptor kinase [60-65].

Sugiyama et al. studied combined treatment in rats after local irradiation with 30 Gy. Recombinant human granulocyte colony-stimulating factor (rhG-CSF), recombinant human macrophage colony-stimulating factor (rhM-CSF) and an inhibitor of transforming growth factor (TGF)-beta1 receptor kinase, were injected into a full-thickness incisional wound site in the dorsal skin. Following combined treatment with the above three compounds the breaking strength of the irradiated skin increased to approximately one-half of that in the non-irradiated skin. Histological analysis of the wounded skin revealed an increase in the formation of collagen fibers. Moreover, the increased breaking strength was associated with an increase in a subpopulation of fibrocytes [60].

## Conclusion

Radiotherapy is an integral modality of head and neck cancer therapy. Compromised wound healing is an important side effect of radiation therapy. The current body of literature comprises a large number of studies investigating the molecular, cellular and clinical effects of compromised wound healing as well as current and possible future therapeutic strategies. Many of the mechanisms leading to cell injuries are still not completely understood. A better understanding of tolerance doses, improved timing of the radiation regimen, and radiation sources would allow a more focused tumor treatment. These advances have led to an improved, but not yet complete protection of healthy tissues. The clinical challenge to optimize wound healing in irradiated patients remains. The present paper critically reviews and summarizes the literature concerning the biology and possibly therapeutic strategies of radiation-induced compromise in wound healing, including stem cell injections and application of growth factors.

## Competing interests

The authors declare that they have no competing interests.

## Authors' contributions

FH and HG had the idea for the literature review and drafted the manuscript. EO prepared figures and tables. FP supported the manuscript by his radio-oncological knowledge. JS and HG contributed through supervision and administrative support. All authors read and approved the final manuscript.
